# Decreased aerobic capacity 4 years after aortic valve replacement in male patients operated upon for chronic aortic regurgitation

**DOI:** 10.1111/j.1475-097X.2011.01072.x

**Published:** 2012-05

**Authors:** Kristofer Hedman, Éva Tamás, Eva Nylander

**Affiliations:** 1 Division of Cardiovascular Medicine, Department of Medical and Health Sciences, University of LinköpingSweden; 2 Department of Cardiothoracic Surgery, Heart & Medicine Centre, LinköpingSweden; 3 Department of Clinical Physiology, Heart and Medicine Centre, LinköpingSweden

**Keywords:** aortic valve insufficiency, cardiopulmonary exercise testing, exercise test, open heart surgery, peak oxygen uptake, physical capacity, physical fitness

## Abstract

Exercise testing is underutilized in patients with valve disease. We have previously found a low physical work capacity in patients with aortic regurgitation 6 months after aortic valve replacement (AVR). The aim of this study was to evaluate aerobic capacity in patients 4 years after AVR, to study how their peak oxygen uptake (peakVO_2_) had changed postoperatively over a longer period of time. Twenty-one patients (all men, 52 ± 13 years) who had previously undergone cardiopulmonary exercise testing (CPET) pre- and 6 months postoperatively underwent maximal exercise testing 49 ± 15 months postoperatively using an electrically braked bicycle ergometer. Breathing gases were analysed and the patients' physical fitness levels categorized according to Åstrand's and Wasserman's classifications. Mean peakVO_2_ was 22·8 ± 5·1 ml × kg^−1^ × min^−1^ at the 49-month follow-up, which was lower than at the 6-month follow-up (25·6 ± 5·8 ml × kg^−1^ × min^−1^, *P* = 0·001). All but one patient presented with a physical fitness level below average using Åstrand's classification, while 13 patients had a low physical capacity according to Wasserman's classification. A significant decrease in peakVO_2_ was observed from six to 49 months postoperatively, and the decrease was larger than expected from the increased age of the patients. CPET could be helpful in timing aortic valve surgery and for the evaluation of need of physical activity as part of a rehabilitation programme.

## Introduction

The natural history of chronic aortic regurgitation (AR) is characterized by insidious development and progression (Bekeredjian & Grayburn, 2005). Symptoms are often unspecific, with patients experiencing fatigue, dyspnoea or intolerance to physical activity. While compensatory mechanisms within the heart limit symptoms, simultaneously the development of irreversible fibrotic changes of the heart muscle may be disguised (Bekeredjian & Grayburn, 2005).

According to current recommendations (Bonow *et al.*, 2006; Vahanian *et al.*, 2007), aortic valve replacement (AVR) is indicated when symptoms have occurred, and may be considered in asymptomatic patients with severe regurgitation. Currently, echocardiography is the predominant method for the evaluation of these patients (Bonow *et al.*, 2006; Katz & Devereux, 2000; Vahanian *et al.*, 2007).

Cardiopulmonary exercise testing (CPET) has been used for a long time in healthy adults and athletes, for determining peak oxygen uptake (peakVO_2_) and physical fitness (Wasserman *et al.*, 1987; Åstrand, 1960). It has proved to be a safe and reliable method for evaluation in various pathologic conditions, including chronic heart failure where it has been found to be of diagnostic, as well as prognostic, value (Ingle, 2008; Myers *et al.*, 2008). Less attention has been paid to the role of CPET in patients with heart valve disease with practice guidelines from 2006 concluding that exercise testing is ‘underutilized’ in these patients. Knowledge is limited concerning changes in peakVO_2_ following AVR in patients with chronic AR.

We have previously found a low aerobic capacity in a group of patients who underwent AVR for chronic AR (Tamas *et al.*, 2009). While postoperative echocardiographic evaluation showed significant improvement in echocardiographic parameters, aerobic capacity remained low 6 months after the AVR. The aim of this study was therefore to investigate peakVO_2_ in patients with chronic AR following AVR, after a longer follow-up time.

## Methods

### Patients

Patients undergoing AVR because of chronic AR between February 2002 and January 2006 at a tertiary centre in Sweden were included in this study. Exclusion criteria were aortic stenosis (defined as an aortic valve area <1·6 cm²) or any other significant heart valve disease at preoperative echocardiographic examination, acute endocarditis or coronary heart disease (the latter based on patient history, physical examination, electrocardiogram, exercise test and previous coronary intervention and, if suspected, verified by coronary angiography).

Twenty-six patients, fulfilling the study entry criteria, who had undergone CPET before and 6 months after their AVR (Tamas *et al.*, 2009) were contacted for a mid-term follow-up. The study was approved by the Regional Ethics Review Board in Linköping, and all enrolled patients gave their written informed consent to participate.

### Cardiopulmonary exercise testing

The testing was carried out with the patients in a sitting position, using an electrically braked bicycle ergometer (E022E; Siemens Elema AB, Upplands-Väsby, Sweden) and with continuous electrocardiographic monitoring (CASE12 12SL Emulation; Marquette Medical Systems Inc., Milwaukee, WI, USA). The patients breathed through an open, low-resistance mouthpiece with their nostrils clamped. Exhaled airflow was measured indirectly by pressure gradients using a linear pneumotachometer (Hans Rudolph Pneumotachometer Model 3800; MedGraphics Corp., St. Paul, MN, USA), and O_2_ and CO_2_ content were analysed on a breath-by-breath basis by two gas analysers (MedGraphics CardiO2 and CPX/D Systems, Spiropharma, Denmark). The pneumotachograph and gas analysers were calibrated prior to each test.

The exercise protocol was chosen individually at the time of the preoperative CPET and consisted of an initial workload of 30–100 Watts for 5–6 min, followed by a continuous increment in workload of 10–20 Watts per minute. Each patient underwent the same protocol at all follow-ups. Patients were instructed to pedal with a constant speed of 60 revolutions per minute until exhaustion. Systolic blood pressure was measured non-invasively every third minute during the test, while perceived exertion, dyspnoea and chest pain were assessed using the Borg scales (Borg, 1982).

### Interpretation of results

We classified the weight-indexed cardiorespiratory fitness of each patient by Åstrand's (1960) and Wasserman's (1987) classifications. For comparison between classifications, patients with a cardiorespiratory fitness in Åstrand's classes denoted ‘high’, ‘good’ or ‘average’ were assigned to the category ‘average or better’, while patients falling into Åstrand's classes ‘fair’ or ‘low’ were categorized as ‘below average’. Furthermore, our patients' peakVO_2_ values were compared with a population-based reference material (Koch *et al.*, 2009).

### Statistical analysis

Data are presented as mean ± standard deviation (SD), and normality was assessed with the Shapiro–Wilk's test. Means were compared with paired *t*-tests, Wilcoxon's signed-ranks test, sign test or McNemar's test, as applicable to the data set. Statistical significance was tested two-sidedly and set to *P* < 0·05. For analyses, SPSS 16.0.1 (SPSS Inc., Chicago, IL, USA) was used.

## Results

### Patients

Patient characteristics are presented in Table 1. Sixteen patients had a mechanical prosthesis implanted, while two received a biological valve and three had aortic valve sparing surgery. No statistically significant correlation between prosthesis type and echocardiographic or cardiopulmonary variables was found (Spearman rho). The second follow-up took place 49 ± 15 months after the AVR and 42 ± 16 months after the first follow-up. Twenty-one of the 26 patients who underwent the first follow-up also completed the second follow-up. Of the remaining five patients, one patient suffered a stroke, one died of cancer, one had leukaemia, one suffered from leg pain and one patient could not be contacted. The patients' regular medication was not withdrawn before the tests and neither did it differ significantly between the two test occasions.

**Table 1:** Patient characteristics.

	Pre-op	6 months post-op	49 months post-op	*P*-value^a^
Age (years)	49 ± 13	49 ± 13	52 ± 12	–
Weight (kg)	86 ± 14	86 ± 14	89 ± 13	0·029
BMI (kg × m^−^²)	27 ± 3	27 ± 3	28 ± 3	0·032
BSA (m²)	2·06 ± 0·21	2·06 ± 0·21	2·10 ± 0·20	0·027
LVEDV (ml)	205 ± 56	123 ± 43	109 ± 30	0·413
LVID (mm)	68 ± 7	55 ± 7	52 ± 7	0·499
EF (%)	54 ± 7	56 ± 10	52 ± 8	0·033

BMI, body mass index; BSA, body surface area; LVEDV, left ventricular end-diastolic volume; LVID, left ventricular internal diameter at end-diastole; EF, ejection fraction at rest.

a *P*-values presented for comparison between postoperative testing.

### Cardiopulmonary exercise testing

Cardiopulmonary exercise testing data are presented in Table 2. Absolute peakVO_2_ was 0·2 ± 0·3 l × min^−1^ (8·3%, *P* = 0·006) lower, and weight-indexed peakVO_2_ was 3·1 ± 3·5 ml × kg^−1^ × min^−1^ (12·0%, *P* = 0·001) lower at the second follow-up. This corresponds to an annual decrease in peakVO_2_ of 2·4% in l × min^−1^ or 3·4% in ml × kg^−1^ × min^−1^ per year. This decrease was larger than expected from the increase in patient age, according to studies of longitudinal changes in peakVO_2_ (Fleg *et al.*, 2005). At this second follow-up, 15 of 21 patients (71%) had a lower peakVO_2_ in l × min^−1^ than at the previous follow-up, while 18 of 21 patients (86%) presented with a decrement in weight-indexed peakVO_2_. The mean ventilatory efficiency (ventilation/carbon dioxide ratio, VE/VCO_2_-ratio) was within normal range (Arena *et al.*, 2008) at all CPETs and not significantly different between postoperative follow-ups. Oxygen pulse was slightly lower at the 49-month follow-up although not statistically significant.

**Table 2:** Cardiopulmonary exercise testing^a^.

	Pre-op	6 months post-op	49 months post-op	*P*-value^b^
Maximal workload (Watt)	184 ± 48	187 ± 40	187 ± 45	0·890
PeakVO_2_ (× min^−1^)	2·2 ± 0·5	2·2 ± 0·5	2·0 ± 0·5	0·006
PeakVO_2_ (ml × kg^−1^ × min^−1^)	26·2 ± 6·6	26·0 ± 5·8	22·8 ± 5·1	0·001
Oxygen pulse (ml × beats^−1^)	14·9 ± 3·1	14·8 ± 3·3	13·5 ± 3·2	0·075
VE/VCO_2_-ratio	28·3 ± 3·2	29·2 ± 3·8	29·6 ± 2·9	0·259
RR (breaths × min^−1^)	30 ± 4	31 ± 6	32 ± 7	0·397
VE (l × min^−1^)	75 ± 15	77 ± 14	76 ± 18	0·874
RER	1·16 ± 0·11	1·17 ± 0·08	1·22 ± 0·10	0·029
Heart rate (beats × min^−1^)	149 ± 18	150 ± 18	152 ± 26	0·828
SBP (mmHg)	207 ± 31	189 ± 31	177 ± 25	0·036

PeakVO_2_, peak oxygen uptake; RR, respiratory rate; VE, ventilation; RER, respiratory exchange ratio; SBP, systolic blood pressure.

a VE/VCO_2_-ratio calculated as mean of values measured at 100%, 75% and 50% of peakVO_2_, all other data recorded at peakVO_2_.

b *P*-values presented for comparison between postoperative tests.

According to Åstrand's classification, all but one patient had a weight-indexed, age-adjusted physical fitness below average (Table 3). No statistically significant differences in number of patients in each category were present between the test occasions. Furthermore, a third of the patients had a peakVO_2_ (ml × kg × min^−1^) that was in the lowest 5% range according to the reference material by Koch *et al.* (2009).

**Table 3:** Number of patients presenting with different levels of physical fitness.

	6 months post-op	49 months post-op	*P*-value^a^
Åstrand (l × min^−1^)			1·000
Average or better	5	4	
Below average	16	17	
Åstrand (ml × kg × min^−1^)			1·000
Average or better	2	1	
Below average	19	20	
Wasserman (l × min^−1^)			0·219
Normal (≥84% of reference)	9	8	
Low (<84% of reference)	12	13	

a *P*-values presented for McNemar's test for repeated measurements of physical fitness between tests.

There was no correlation between systolic left ventricular function at rest (i.e. ejection fraction) and peakVO_2_ at the 49-month follow-up (*r* = 0·083, *P* = 0·728). Furthermore, no significant differences for echocardiographic variables were found between patients with ‘low’ and ‘normal’ physical fitness according to Wasserman's classification.

## Discussion

Reports of peakVO_2_ changes in patients with AR following AVR are scarce (Kim *et al.*, 2003; Marino *et al.*, 2006; Trikas *et al.*, 1994), and the results for patients with different valvular pathologies are seldom presented separately. Moreover, not all studies have determined peakVO_2_ but rather examined changes in exercise capacity in Watts (Gohlke-Bärwolf *et al.*, 1992; Niemelä*et al.*, 1983).

After reporting previous findings (Tamas *et al.*, 2009) of unchanged peakVO_2_ in patients with chronic AR 6 months following AVR, we now sought to study more long-term changes in peakVO_2_ in the same patients. Interestingly, the present study revealed a significant decrease in peakVO_2_ since the previous follow-up, both in absolute (ml × min^−1^) and weight-indexed (ml × kg^−1^ × min^−1^) measures, which rules out that the decrement was solely an effect of the increased body mass of the patients at the late follow-up.

This is in contrast with the results of Niemelä*et al.* (1985), who found no difference in peakVO_2_ between patients and healthy controls 1 year after AVR, and in a subgroup of patients, peakVO_2_ was significantly higher postoperatively than preoperatively.

Two studies (Kim *et al.*, 2003; Trikas *et al.*, 1994) on patients with mixed valvular lesions have revealed postoperative improvement in aerobic capacity, at 6 and 12 months, respectively. Further two studies (Gohlke-Bärwolf *et al.*, 1992; Niemelä*et al.*, 1983) demonstrated an increase in exercise capacity, indicating increased aerobic capacity after surgery, while one study (Marino *et al.*, 2006) on patients operated upon with the Ross procedure showed no postoperative change in peakVO_2_. To our knowledge, deterioration in peakVO_2_ following surgery has not been described by others, although the follow-up period in the present study was longer than seen elsewhere.

Several factors that affect aerobic capacity may contribute to this difference and need to be taken into consideration. First, the data from early and late follow-up must be comparable, which was confirmed by similar respiratory exchange ratios, well above one, at both CPETs (Table 2).

Second, peakVO_2_ decreases with age (Fleg *et al.*, 2005; Koch *et al.*, 2009; Wasserman *et al.*, 1987; Åstrand, 1960), and our patients were, on average, three and a half years older at the second follow-up (Table 1). However, the decrease in aerobic capacity in our patients was larger than expected just by their increased age (Fleg *et al.*, 2005). The majority of patients were still of a physical fitness level that was below average according to age-indexed reference values (Table 3), and a third of patients had a peakVO_2_ in the lowest 5% range as calculated by algorithms from Koch *et al.* (2009).

The majority of our patients were on cardiac medication, including β-blockers, with no significant differences in reported use of any medication between follow-ups. Although β-blockers decrease maximal heart rate during work, it is unclear whether, and to what extent, they reduce physical work capacity in patients with AR. In patients with coronary artery disease, peakVO_2_ remains unchanged owing to peripheral compensatory mechanisms (Eynon *et al.*, 2008).

One of the important determinants of peakVO_2_ in healthy individuals is the level of physical activity (Wasserman *et al.*, 1987; Åstrand, 1960). The participating patients were inhabited in the geographically fairly large region of south-east Sweden, and rehabilitation was taken care of by the referring hospitals. Their attendance to a cardiac rehabilitation programme, or general physical activity, was not recorded within the frames of the present study. However, all patients were encouraged to resume regular physical activity at discharge. It is possible that the decrease in peakVO_2_ in our patients was, at least in part, attributable to a more sedentary lifestyle postoperatively. Becassis *et al.* (2000) found no significant difference in peakVO_2_ between a group of healthy controls and patients who had received aortic heart valve prosthesis 1 year postoperatively. This could indicate that the heart valve or the open heart surgery *per se* did not affect peakVO_2_. However, of the patients in their study, only one presented with AR preoperatively, and the patients were considerably smaller than in the current study and therefore had lower reference values than our patients.

Finally, a subnormal postoperative left ventricular function cannot be ruled out despite the normalization of the recorded echocardiographic parameters, which were comparable in Wasserman's classes ‘low’ and ‘snormal’, and without statistically significant in-between group differences.

### Cardiac rehabilitation and peakVO_2_ following aortic valve replacement

In contrast to the solid evidence for beneficial effects of exercise training in patients with coronary artery disease (Hansen *et al.*, 2005; Wenger, 2008; Williams *et al.*, 2006) and chronic heart failure (Davies *et al.*, 2010), knowledge is limited when it comes to exercise training in rehabilitation of patients with AR, following AVR. The few studies addressing this matter all include heterogeneous groups of patients regarding their valvular lesions (Jairath *et al.*, 1995; Landry *et al.*, 1984; Newell *et al.*, 1980; Sire, 1987; Ueshima *et al.*, 2004).

In studies published more than 20 years ago (Landry *et al.*, 1984; Newell *et al.*, 1980; Sire, 1987), a positive effect on aerobic capacity was seen with training programmes ranging from four to 24 weeks in length, when compared to control groups. In contrast, more recent studies failed to find any effect on peakVO_2_ with a three (Jairath *et al.*, 1995) or six (Ueshima *et al.*, 2004) months training programme. A recent case study showed that a single patient undergoing AVR for AR responded well to a large volume of regular exercise and not only tolerated it but also almost doubled peakVO_2_ and normalized left ventricular function, assessed by echocardiography, within 1 year (Pressler *et al.*, 2011).

The conflicting results from previous studies may possibly be explained by diversity in length or character of the training protocols studied. Unfortunately, details of training programmes are sparsely presented, and no study compares different training modalities, making it troublesome to recommend a certain exercise regimen. Furthermore, the groups studied are often heterogenic, with several valvular pathologies intermixed. The date of the studies ranges from 1980 (Newell *et al.*, 1980) to 2004 (Ueshima *et al.*, 2004), which could be of relevance because recommendations for timing of surgery have changed over the years, towards earlier intervention, and surgical techniques have, in parallel, developed further.

### Conclusion and clinical implications

A significant decrease in peakVO_2_ was observed from six to 49 months postoperatively, in patients operated upon for chronic AR representing an impaired aerobic capacity. Using CPET as part of the preoperative screening and follow-up could be helpful in timing aortic valve surgery and for the evaluation of need of physical activity as part of a rehabilitation programme aiming at decreasing morbidity and improving quality of life in patients with AR undergoing AVR.
